# Intra-Phenotypic and -Genotypic Variations of *Beauveria bassiana* (Bals.) Vuill. Strains Infecting *Aedes aegypti* L. Adults

**DOI:** 10.3390/ijms25168807

**Published:** 2024-08-13

**Authors:** Norma Zamora-Avilés, Alonso A. Orozco-Flores, Teodora Cavazos-Vallejo, César I. Romo-Sáenz, David A. Cuevas-García, Ricardo Gomez-Flores, Patricia Tamez-Guerra

**Affiliations:** 1 Departamento Ecología de Artrópodos y Manejo de Plagas, El Colegio de la Frontera Sur (ECOSUR), Carretera Antiguo Aeropuerto Km 2.5, Tapachula 30700, Mexico; norma.zamora@ecosur.mx; 2 Laboratorio de Inmunología y Virología, Facultad de Ciencias Biológicas, Universidad Autónoma de Nuevo León, San Nicolás de los Garza 66455, Mexico; aorozcof@uanl.edu.mx (A.A.O.-F.); teodora.cavazosvl@uanl.edu.mx (T.C.-V.); cesar.romosnz@uanl.edu.mx (C.I.R.-S.); david.cuevasgrc@uanl.edu.mx (D.A.C.-G.); rgomez60@hotmail.com (R.G.-F.)

**Keywords:** introns profile, dengue vector, entomopathogenic fungus, strain detection

## Abstract

*Beauveria bassiana* has potential for *Aedes aegypti* biological control. However, its efficacy depends on the strain’s geographic location, host susceptibility, and virulence. The present study aimed to evaluate the effectiveness of *B. bassiana* strain BBPTG4 conidia in controlling *Ae. aegypti* adults and its detection via introns profile on exposed mosquito corpses. Morphologic characteristics among strains were highly similar. Comprehensive testing of these strains demonstrated that BBPT4 exhibited the ideal biological activity for *Ae. aegypti* control, with a median lethal time (TL_50_) of 7.5 d compared to ~3 d and ~10 d for BB01 and BB37 strains, respectively. Infected mosquitoes died after GHA and BBPTG4 exposure, and corpses were analyzed for infecting strains detection. Differences among the seven evaluated strains were determined, assessing five different insertion group I intron profiles in BBTG4, BB01, GHA, BB37, and BB02 strains. Mosquitoes infected by BBPTG4 and non-exposed (negative control) intron profiles were obtained. We detected the presence of introns in the BBPTG4 strain, which were not present in non-exposed mosquitoes. In conclusion, *B. bassiana* strains showed similarities in terms of their cultural and microscopic morphological characteristics and biologicals virulence level, but different intron profiles. BBPTG4 strain-infected *Ae. aegypti* adult corpses, showing specific amplicons, enabled us to identify *B. bassiana* at the strain level among infected mosquitoes. However, monitoring and detection of field-infected insects is essential for further verification.

## 1. Introduction

In recent decades, *Aedes aegypti* L. (Diptera: Culicidae) has emerged as a significant urban pest with the highest transmission of viral diseases, such as dengue, zika, chikungunya, and recently the Mayaro fever [1]. An increasing global death toll resulting from the transmission of these arboviruses has been observed. In 2023, cases of dengue in Mexico were estimated at 54,406 and 69,437 by the Mexican Ministry of Health (Secretaría de Salud Pública (SSP)) [2] and the Pan American Health Organization (PAHO) (PAHO/WHO) [3], and the SSP estimated the cases of zika and chikungunya in 58 [4] and 4 [5], respectively. Currently, the predominant approach for controlling *Ae. aegypti* involves the extensive use of chemicals pesticides such as temephos and pyrethroids, standing as the primary choice for managing vector populations [6]. However, negative biological and environmental effects have emerged, including mosquito-resistant selection, the presence of secondary pests, and a decline in beneficial populations [7].

Biological control methods show alternatives for resisting this vector, thereby mitigating the adverse effects of chemical insecticides and enhancing the ecosystems with biodegradable and ecologically friendly options [8]. The efficacy of entomopathogenic fungi (EPF), including *Metarhizium* spp. and *Beauveria* spp., has been reported against *Ae. aegypti* and other genera of the Culicidae family [9]. *Beauveria bassiana* (Bals.) Vuillemin is a cosmopolitan fungus reported as a tool for these vectors’ control. Nevertheless, *B. bassiana* strain’s virulence may vary according to its origin, genetic characteristics, and infectivity against tested or target mosquitoes [10]. For instance, after analyzing 29 *B. bassiana* isolates with varying virulence against the malaria-transmitting mosquito *Anopheles gambiae*, Coetzee, Hunt, and Wilkerson underscored the importance of carefully selecting EPF strains for effective insect control [11]. 

The importance of detecting and demonstrating the effectiveness of *B. bassiana* strains in the laboratory, greenhouse, and field has been recognized [12]. However, it is challenging to identify the morphological characteristics of *B. bassiana* strains. In addition to laborious procedures of strain isolation under laboratory conditions in culture media from infected insects, at least 10 d are required for culturing strains [13]. Furthermore, molecular techniques by which to identify EPF strains infecting dead insects in laboratory and field conditions are needed to demonstrate their efficiency. Thus, molecular tools may help us to identify specific isolates from infected corpses [14] and demonstrate that the aerial mycelium developed on the insect corpse was killed by a specific *B. bassiana* strain. Traditionally, the use of ribosomal DNA genes’ (rDNA) internal transcribed spacers (ITS) has been described for identifying EPF [15,16], but this technique only detects the genus and species and does not discriminate between isolates. Techniques such as randomly amplified polymorphic DNA (RAPDS) are common for *B. bassiana* strain discrimination [17,18] but have poor reproducibility, basically due to the use of short primers under standardized conditions. For RAPDS, the complex patterns obtained may have different interpretations; it has to be performed under axenic isolation, and it is uncertain as to the site where the primers will bind [19]. Another approach is using sequencing characterized amplified region (SCARS), which has shown appropriate results, but this technique requires sequencing, where specific primers must be designed for every tested strain [20]. Thus, SCARS is usually an expensive and time-consuming analysis. Furthermore, fungal microsatellites are more difficult to isolate, and fungi exhibit lower polymorphism than other organisms [21].

However, we need a tool for genetic intraspecific differentiation—for instance, PCR amplification of conserved regions between species. Regarding group I introns (GIi), they are erratic and are only present in some fungal strains and absent in others [22]. GIi are ribozymes that catalyze their splicing and are present in rRNA, tRNA, and ribosomal coding genes of plants, fungi, lower eukaryotes, bacteria, and eubacteria genomes [23]. Four intron insertion sites have been detected in this group in the 3′OH of the 28S DNA gene from *Beauveria* species, and insertion pattern differences between this intron have been useful for identifying different *B. brongniartii* [24] and *B. bassiana* strains [25]. More recently, Cavazos-Vallejo et al. [26] reported a molecular sensible technique for endophyte *B. bassiana* detection in bean plants, which used 10 ng of rDNA for a nested PCR detecting the different profiles of GIi in the 28s gene of a commercial strain (GHA) and the *B. bassiana* strains BB37 and BBPTG4, which are used in this study.

The present study aimed to assess the macro morphological characteristics of six strains and their virulence levels against *Ae. aegypti* adults. In addition, we discriminated between *B. bassiana* strains by analyzing the insertion patterns of group I introns in six pure strains, one strain obtained from a commercial product (GHA), and one strain directly detected in dead *Ae. aegypti* adults intentionally infected under laboratory conditions. Furthermore, one of the strains was selected for future biological control of *Ae. aegypti* adults, and we detected its molecular characteristics in the field to demonstrate its efficacy in a mosquito control strategy.

## 2. Results

### 2.1. Macroscopic Morphological Description of Beauveria basssiana Strains 

Under laboratory conditions, all tested *B. basssiana* strains shared the characteristic beige/yellowish colony color on both sides except for BBPTG4, which developed a pink-reddish pigment on the lower side of the PDA plate. BBPTG4 and BBPTG6 strains showed morphological similarities such as color (beige), shape (circular), elevation (convex), and days for colonies covering the dish surface (6 d) (Supplementary Table S1).

Strains showed differences in growth pattern and texture, where BBPTG4 grew sparser than BBPTG6 and was velvety in appearance. The only strain that presented filiform and dispersed growth patterns was the BB37 strain (Supplementary Table S1). In general, strains from agriculture fields showed similarities in color and growth pattern, as compared with the BBPTG6 strain. Differences detected regarding texture showed that GHA, BB37, and BB42 strains had a colony texture similar to that of BBPTG6, whereas BB01 and BB02 strains had a velvety texture similar to that of BBPTG4. Regarding colony-mycelia elevation, BB37 strains were similar to agriculture field isolated strains, whereas all other strains produced mycelia-raised elevation (Supplementary Table S1).

### 2.2. Virulence (LT_50_) of B. bassiana Strains against Ae. aegypti Adults

After testing *Ae. aegypti* adults exposed to *B. bassiana* strains, the mosquito population was reduced to up to 69.5%. In general, strains were infective and virulent against this important vector, resulting in a LT_50_ average of 5.6 d (Table 1). BB37 strain had the shortest LT_50_ (2.92 d) and higher relative potency, as compared with all other strains. According to the overlap of the estimated confidence limits for each strain, BB02, GHA, and BBPTG6 strains showed similar LT_50_, ranging from 4 d to 5 d, to LT_50_ in *Ae. aegypti* adults. The LT_50_ of BB01 strains was the highest, whereas the selected strain for molecular evaluation (BBPTG4) showed an LT_50_ of two days less than that of BB01 (Table 1). 

### 2.3. Subunit I Intron Amplification from DNA of B. bassiana Strains

**Figure 1:**
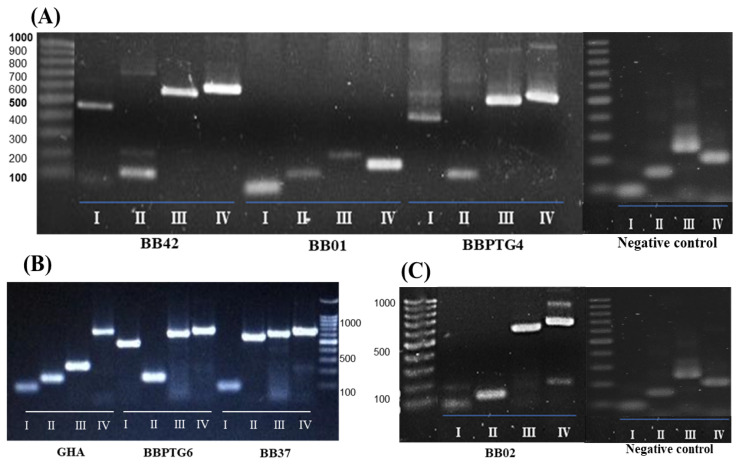
PCR introns insertion profiles (I–IV) for six Mexican and one commercial (GHA) *Beauveria bassiana* strains, where the negative control shows PCR introns insertion profiles (I–IV) from rDNA samples of *Aedes aegypti* adults unexposed with *B. basssiana*. Molecular markers of 100 bp. (**A**) DNA introns profile (I–IV) from BB42, BB01, and BBPTG4 *B. basssiana* strains, and uninfected *Ae. aegypti* adults (negative control); (**B**) DNA introns profile (I–IV) from GHA (positive control), BBPTG6, and BB37 *B. basssiana* strains; (**C**) DNA introns profile (I–IV) from BB02 *B. basssiana* strain and uninfected *Ae. aegypti* adults (negative control).

The introns profile results and each strain’s information are shown in Figure 1. The results of the DNA large-subunit introns (I–IV) of strains were compared between strains, showing strains differentiation as specific markers and a similar pattern between BB42, BBPTG4, and BBPTG6 (I1P, I2A, I3P, and I4P).

The following intron profiles were obtained: BB42, BBPTG4, and BBPTG6 showing introns I (501 bp), II (157 bp), III (606 bp), and IV (620 bp). The BB01 strain had amplification fragments of ~84, 157, 244, and 207 bp, without insertion of introns at any position (I1A, I2A, I3A, and I4A). The BBPTG4 strain showed amplifications of ~501, 157, 606, and 620 bp, respectively (I1P, I2A, I3P, and I4P) (Figure 1A). In addition, the GHA strain showed fragments of ~84, 157, and 244 for the amplification of the insertion sites I, II, III, and IV, showing that only intron IV was inserted (~620 bp) (I1A, I2A, I3A, and I4P). 

Furthermore, the BBPTG6 strain amplified fragments that showed no insertion of intron II, with a 157 bp size (Figure 1B), whereas rDNA from the BB37 strain resulted in three insertions corresponding to introns II, III, and IV with 656, 606, and 620 bp (I1A, I2P, I3P, and I4P), respectively (Figure 1B).

The BB02 strain’s DNA amplified fragments were of ~84, 157, 606, and 620 bp (I1A, I2A, I3P, and I4P), thus indicating that only introns in positions III and IV were present (Figure 1C). These results demonstrated genetic variability between the different strains studied since intron IV was present in 91% of the strains, whereas introns I, II, and III were present in 42.8%, 14.3%, and 71.4% of tested strains, respectively.

### 2.4. BBPTG4 and GHA Introns Profile in Ae. aegypti Adults

**Figure 2:**
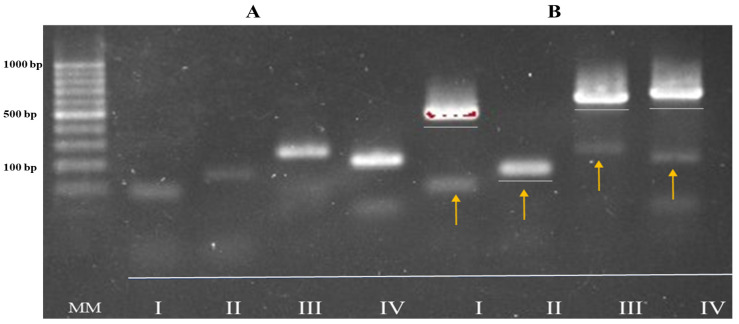
PCR introns insertion profiles (I–IV) from *Aedes aegypti* adults rDNA samples uninfected or infected with *Beauveria basssiana* BBPTG4 strain. Promega molecular marker of 100 pb for (**A**,**B**) images. (**A**) DNA introns profile (I–IV) from uninfected *Ae. aegypti* adults and (**B**) *Ae. aegypti* adults infected with BBPTG4 *B. bassiana* strain. Grey lines represent the introns profile of the BBPTG4 *B. bassiana* strain, and the yellow arrows show the profile of insertion sites of introns of *Ae. aegypti* DNA.

After analyzing the introns profile sites from BBPTG4 culture mycelium DNA, we amplified the bands of ~ 501, 157, 606, and 620 bp, corresponding to I1P, I2A, I3P, and I4P profile (Figure 1A,C, and negative control), which we directly detected in mosquito adults. In the case of mosquitoes, the uninfected DNA showed a profile with an absence of introns in the insertion sites 84, 157, 244, and 207 bp for I1A, I2A, I3A, and I4A, respectively (Figure 1A,C and Figure 2A). 

The introns profile developed by BBPTG4 trap-infected *Ae. aegypti* from randomized selected mosquitoes was the same as that observed in the mycelium from PDA culture. After evaluating the *Ae. aegypti* DNA introns profile from contaminated laboratory adults, we showed both profiles for BBPTG4 and mosquitoes, thus demonstrating the infection in mosquito’s corpses for the strain selected (I1P, I2A, I3P, and I4P) (Figure 2B).

## 3. Discussion

*B. bassiana* species are widely distributed, which underscores the significance of comprehending its intraspecific morphological, virulence activity, and genetic characteristics. This knowledge is crucial for harnessing its potential in agricultural and urban pest biocontrol [27,28]. 

In this study, the macro morphological characteristics, the virulence of *B. bassiana* strains against *Ae. aegypti*, and the intron profiles of several *B. bassiana* strains and of *Ae. aegypti* were determined. The effectiveness of the *B. bassiana* strain BBPTG4 conidia to control *Ae. aegypti* adults was assessed and its detection via introns profile on exposed mosquito corpses was achieved. 

Our macro morphological study revealed similarities to *B. bassiana* strains. Evaluated strains were selected because they did not immediately kill *Ae. aegypti* adult mosquitoes to allow them to spread the pathogen. All tested strains exhibited uniform traits, including color, growth pattern, and shape among others, posing challenges for their distinct identification, except BBPTG4, which showed a pink-reddish pigment on the lower side of the PDA plate and, in some cases, colony growth deficiencies (BB01, BB02, and BB42, Supplementary Table S1). 

Despite originating from different isolation sources, including agriculture soil sites and cockroaches (Supplementary Table S1), our study agrees with other reports where *B. bassiana* strains were obtained from various environments such as alpine soil, heathland, peat bogs, soil with savannah-type vegetation, and forest soil among others, as shown by *B. bassiana* samples from six different localities in Ethiopia [29,30].

Moreover, Uztan et al. [16] identified 32 positive sites for *B. bassiana* strains in Turkey, revealing both micro and macro morphology similarities. Other studies have highlighted the challenges associated with identifying *B. bassiana* species based on micro-phenotypic characteristics such as the shape and size of conidia [31]. Due to these difficulties, molecular techniques are required to be implemented. The SCAR and RAPD methods have been applied, albeit with certain complexities [18,20]. The widely utilized region 28s of rDNA, known for identifying fungal genera, particularly incorporates group I intron insertion sites for major intraspecific definition [25,32]. 

These molecular approaches allow for the differentiation of five out of seven *B. bassiana* strains, including BBPTG4, BB01, BB02, and BB37, as well as the commercial strain GHA. Although BB42 and BBPTG6 share a similar introns profile to that of BBPTG4, it exhibits a distinctive macro-morphological feature, characterized by the development of a pink-reddish pigment and optimal conidia production. 

Similar results were reported by Wang et al. [25], who demonstrated that 125 *B. bassiana* strains had a distribution and size variation on the intron profile. Another study showed the same four intron profiles as in our study, where the BB37 strain showed intron I to be absent and II–IV to be present; the GHA strain showed the absence of introns I–III and the presence of intron IV; and in the BBPTGA strain, intron II was absent but introns I, II, and IV were present. In addition, we obtained, for the first time, the BB02, BB01, BB47, and BBPTG6 strains’ intron profiles [26].

The intron profile of BBPTG4 was identified in infected *Ae. aegypti* adult cadavers, which differed from that detected in uninfected mosquitoes. Following the assessment of treatment efficacy against *Ae. aegypti*, we demonstrated the infection of mosquitoes by the BBPTG4 strain using PCR protocols, as reported for the identification of vegetal tissues [26,32]. The analysis of the intron profile revealed regions not detected in the *Ae. aegypti* sample, with only the insert regions of introns showing fragments of less than ~300 bp.

Differences in pathogenicity among *B. bassiana* strains have been previously explored [33], demonstrating the entomopathogenic potential of this species against adults of *Ae. aegypti* and emphasizing that certain strains may induce up to 70% cumulative mortality in mosquito populations after 7 d of conidia exposure. In the present study, a concentration of 1 × 10^8^ conidia/mL showed >50% mortality in most cases, except for BB37, with <40% mortality. However, the biological effectivity of a strain is determined by the LT_50_, wherein the most aggressive and virulent is characterized by its potential to eliminate its host in the shortest time (d), as in a *B. bassiana* study for the dipteran fruit fly in the laboratory [34]. 

After analyzing our data, the calculated LT_50_ ranged from 2.9 d to 9.7 d. However, other authors have examined even more virulent strains with a mean lethal time of 2.70 d to 5.33 d, demonstrating LT_50_ values lower than those reported in this study [35]. When targeting different species as dipterans, such as *Anastrepha ludens* (Loew.), the reduction of the population by half has been observed from 2.8 d to 5.99 d [34]. In contrast, certain species within the *Beauveria* genus, such as *B. brongniartii* isolated from lepidopteran species, showed higher LT_50_ values against *Ae. aegypti*, reaching 11 d [36]. The variation in the time required to reach 50% insect mortality arises due to several factors, with EPF strain virulence being one of the main factors, along with the host species from which the pathogen was isolated and the age and sex of the host being evaluated, in addition to environmental conditions [29,37]. Previous reports indicate that the susceptibility of insect hosts towards a specific entomopathogen presents differences between colonies or insect populations due to natural selection and environmental conditions, with variation being attributed to the prolonged biological association between host and pathogen over the years inside a niche [38]. 

Several *B. bassiana* strains have shown their potential to control *Ae. aegypti*, but in a different stage of its life cycle and mostly by using chemicals. Therefore, this EPF represents a viable alternative with which to fight this important vector, without the risk of affecting the environment and damaging health. All *B. bassiana* strains infected and killed up to 50% of the exposed mosquito population in just over 7 d. Notably, the BBPTG4 strain had the best characteristics, having an LT_50_ that allows the mosquito to transmit conidia to other adults for a few days until its eventual death. 

## 4. Materials and Methods

Reagents were obtained from Sigma-Aldrich Química, S.A. de C.V. (Toluca de Lerdo, Mexico) unless otherwise specified.

### 4.1. Mosquito Source and Rearing Conditions

*Aedes aegypti* L. strain was provided by the Laboratorio de Entomología of Facultad de Ciencias Biológicas at Universidad Autónoma de Nuevo León (UANL), México. *Ae. aegypti* colony was kept inside an insect breeding room at 28 °C and 80% relative humidity (RH), following the protocol described in the guide for the installation and maintenance of *A. aegypti* (Diptera: Culicidae) insectary (http://www.cenaprece.salud.gob.mx/programas/interior/vectores/descargas/pdf/GuiaInstalacionMantenimientoInsectario.pdf (website accessed on 5 March 2023)) from the Ministry of Health of Mexico. 

For this, adults were kept in a 38.1 cm width, ×60.0 cm height pop-up butterfly cage (Carolina Biological Supply Company, Burlington, NC, USA), placed inside an insect rearing room at 25 °C ± 2 °C and 60% ± 10% RH with a 14 h:10 h light–darkness photoperiod. Mosquitoes were fed on 5% sucrose-solution-soaked cotton balls in a 20 mL plastic cup placed near one of the cage corners. Sucrose solution was daily replenished using a 3 mL syringe, whereas females were also fed blood from a human arm, following the protocol. For oviposition, 2 L plastic cylindrical containers with 700 mL of tap water and 0.5 g of fish flakes (Wardley, Grupo Acuático Lomas, S.A. de C.V., Cuajimalpa, Mexico) were placed inside the adult cage [11]. 

After larvae emerged, the container top was covered with muslin mesh and the oviposition container was replaced with a new one. The larvae, from neonate to fifth instar, were fed with fish flakes. Pupae were transferred to cages for adult emergence, and this cycle was repeated. The emerged 5- to 8-d-old *Ae. aegypti* adults were transferred to a different release cage for bioassays. About 20 males and 20 females were kept untested for maintaining the mosquito colony under the rearing conditions described above.

### 4.2. B. bassiana Strains Source and Phenotypic Characterization

*Beauveria bassiana* (Bals.) Vuillemin strains were provided by the Laboratorio de Inmunología y Virología in the Unidad de Formulación de Biológicos (UFB) at UANL, Mexico. BBPTG4 (Genbank: KC759730), and BBPTG6 strains were isolated from *Periplaneta americana* L., collected in San Nicolás de los Garza, Nuevo León, Mexico (25°45′00″ N 100°17′00″ O). BB01, BB02, BB42, and BB37 strains were provided by the Comité Estatal de Sanidad Vegetal, Guanajuato, Mexico, from agricultural soils. In addition, the GHA commercial strain (Botanigard, Certis Biologicals, Columbia, MD, USA) was used as a reference *B. bassiana* strain. Strains were maintained in the Colección de Hongos Entomopatógenos in the UFB at UANL.

To characterize the *B. bassiana* strains macro phenotypes, they were grown on potato dextrose agar (PDA) (BD Difco, Ciudad de México, Mexico) in Petri dishes (5 cm diameter × 1 cm depth) (Med Lab S.A. de C.V., Ciudad de México, Mexico), incubated at 25 °C ± 2 °C until sporulation was observed. Each strain was morphologically identified to confirm that they presented the same phenotype as described after their isolation, including mycelia color, growth pattern, shape, texture, elevation, and days required for conidia production [39].

For conidia reactivation, strains were grown on PDA from a monosporic stock storage in mineral oil. Fungus inoculation was made with 100 µL of this stock sample in oil. Inoculated Petri dishes were then incubated at 25 °C for 5 d to 7 d in darkness until sporulation was observed [40].

### 4.3. B. bassiana Strains Virulence against Ae. aegypti Adults

For semi massive production, the conidia stock was inoculated into an Erlenmeyer flask with 200 mL of potato dextrose broth (PDB) (BD Difco). Culture was further incubated at 25 °C ± 2 °C in an automatic rotary shaker at 120 rpm (Orbit1900; Labnet, Ciudad de México, México) for 5 d until blastopores structures were detected and adjusted to 1 × 10^8^ blastospores/mL using a Neubauer chamber under a phase-contrast microscope at 40× [40,41]. For bioassays, *Ae. aegypti* adults were infected under laboratory conditions. 

Strain blastospores were used to inoculate rice for conidia production via solid fermentation. In brief, 100 g of pre-moistened sterile parboiled rice grains, used as the solid substrate, were placed in 800 mL glass bottles, containing 30 mL of hydration sterile solution (0.97 g/L KH_2_PO_4_, 410 µL/L H_2_SO_4_, and 0.31 g/L yeast extract). Bottles containing moistened rice were autoclaved for one hour. During incubation, rice solid culture in bottles was daily mixed with a spatula for aeration under sterile conditions. 

Rice-cultured conidia were harvested using a standard No. 40 sieve (426 µm pore size). Produced conidia were quantified by taking a 15 mg sample, suspending it in 0.5% INEX-A (emulsifier agent), counting, determining germination counting, and storing at 4 °C to prepare different concentrations for biological activity testing [39]. 

Twenty microliters of this suspension were inoculated on PDA and incubated at 25 °C for germination. Germination percentage was then determined by counting 100 conidia and determining the amount of those showing the germinative tube, in triplicate, to determine the conidia germination percentage [39]. 

The median lethal time (LT_50_) was analyzed among *B. bassiana* strains, except BB42, for growth deficiencies. We prepared a homogeneous stock solution of 1 × 10^10^ conidia/mL diluted in 0.5% INEX-A for semi-massive production. Conidia were then adjusted using 0.5% INEX-A solution to a final concentration of 1 × 10^8^ conidia/mL, a selected concentration that induced ~90% mortality, in a final volume of 1.9 mL. We then exposed 20 mosquito adults to conidia in triplicate. The conidia suspension was dispersed on previously sterilized filter paper layer, which was placed inside the cylinder at the container base in addition to cotton wool soaked in 5% sugar solution for mosquito feeding. Bioassay conditions were the same as for the rearing conditions (25 °C ± 2 °C, 60% ± 10% RH, and 14 h:10 h light–darkness photoperiod), where mosquitoes were fed on 5% sucrose solution-soaked cotton balls. After 48 h of mosquitoes’ exposure to six strains or sterile distiller water, they were transferred to new containers with 250 mL capacity, each one with sufficient food to ensure their survival, following the method described by Castrejón-Antonio et al. [41]. Survival and infection monitoring was conducted every 12 h for 15 d. Dead mosquitoes were transferred to a humid chamber to detect aerial mycelium development from corpses to confirm the mortality due to *B. bassiana* [40]. LT_50_ values were estimated using time–mortality data, where the analysis was performed using a lineal regression model (Polo Plus, V. 1.0) [41]. 

### 4.4. Molecular Characterization of Seven B. bassiana Strains 

#### 4.4.1. DNA Extraction

Genomic DNA extraction was performed from PDB culture fresh mycelium from each strain after incubating at room temperature for 5 d at 120 rpm (Labnet International, Cary, NC, USA). After this, 0.1 g of the developed submerged mycelium was frozen at −70 °C, from which we extracted DNA. We used the Wizard Genomic Purification DNA extraction kit (Promega Corp., Madison, WI, USA), according to the manufacturer’s protocol A1125 with some modifications. Centrifuged pellet samples were washed with sterile distilled water, after which 600 µL of nuclei lysis solution were added, and the mixture was macerated with a pistil, vortexed for three seconds, and incubated at 65 °C/15 min. Next, three microliters of RNAse solution were added, and the tube was inverted 2 to 5 times and incubated for 15 min at 37 °C and 5 min at room temperature. We then added 200 µL of protein precipitation solution with a 20 s vortex step and centrifuged it at 14,000 rpm for 10 min. The supernatant was then transferred to a new tube with pure isopropanol for DNA precipitation (1:1); the tube was inverted 4 to 5 times and centrifuged at 14,000 rpm for 10 min at room temperature. Isopropanol was discarded, and the pellet was washed with 70% ethanol and centrifuged at 14,000 rpm for one minute. The pellet was air-dried, and the DNA was dissolved in 25 μL of sterile milli-Q water and quantified via spectrophotometry using a NanoDrop Lite Spectrophotometer (Thermo Fisher Scientific Inc., Waltham, MA, USA).

#### 4.4.2. Profile of *B. bassiana* Strains by PCR 

Four individual PCR reactions were made per strain to determine the intron insertion pattern for the BBPTG4, BBPTG6, BB01, BB02, BB37, BB42, and GHA *B. bassiana* (BB) strains analyzed. In addition, four Group I intron insertion sites in the 3′OH region of the ribosomal DNA large subunit were amplified [7] to determine if the strains had distinct intron insertion patterns.

Only the reverse primer sequence of intron I was changed from that previously reported [33,34]. The primers used to verify the introns profile insertions were as follows: E23 F (5′-CCG AAG GAA TTC GGT AAG CG-3′) and M1R (5′-GGT AAA ACT AAC CTG TCT CAC G-3′) for intron I; I21 F (5′-CGA TCC TTT AGT CCC TCG AC-3′) and I22 R (5-CGC TTA CCG AAT TCC TTC GG-3′) for intron II; I38 F (5′-ATG GGC TTG GCA GAA TCA GCG-3′) and I32 R (5′-CAG CCA AAC TCC CCC CCT G-3′) for intron III; and I29 F (5′-CTG CCC AGT GCT CTG AAT GTC-3′) and I31 R (5′-CGC TGA TTC TGC CAA GCC CAT-3′) for intron IV. The reaction consisted of (final volume 20 μL) 1 μM of each primer (reactions per intron I, II, III, and VI were separated), 1 μL of fungus DNA (concentration 100 ng/μL), and 20 μL of GoTaq^®^ Green Master Mix (Promega Corp.), with a cycling program of 4 min at 95 °C as an initial denaturation step, followed by 35 cycles of 45 s denaturation at 94 °C, 45 s annealing at 57 °C, 45 s extension at 72 °C, and a final extension step of 4 min at 72 °C. 

For the amplifications, and if the intron is present (P) in the strain, the gel is expected to show a heavier band; if the intron is absent (A), the gel is expected to show a lighter band. For instance, by intron I, a 501 pb fragment develops if the intron was P, or an 84 pb fragment develops if the intron was A. For intron II, a 656 pb fragment develops if the intron was P, or a 157 pb fragment develops if the intron was A. Furthermore, for intron III, it was stated that the presence of a 606 pb fragment indicates that the intron was P, but the presence of a 244 pb fragment indicates that intron was A. Similarly, for intron IV, the presence of a 620 pb fragment indicates that the intron was P, whereas the presence of a 207 pb fragment indicates that the intron was A. The four amplification of DNA product bands were obtained in 1% agarose gel at 119 V for 90 min, stained with ethidium bromide, and visualized in an UV trans-illuminator. The commercial GHA strain mycelium was evaluated as positive control. 

In order to detect any similarities or differences with the *B. bassiana* intron profile, the intron profile of *Ae. aegypti* adults was analyzed and tested as a negative control. Genomic DNA extraction was performed on three corpses of mosquitoes. DNA unexposed to *B. bassiana* was extracted using the Promega Wizard Genomic Purification DNA kit. The primers used to verify the introns’ insertions profile, using the same conditions for intron amplification reactions as described above, were the following: E23 and M1R for intron I; I21 and I22 for intron II; I38 and I32 for intron III; and I29 and I31 for intron IV.

### 4.5. BBPTG4 Strain Detection in Ae. aegypti Adult Corpses

We evaluated the BBPTG4 strain for molecular detection in mosquito adults based on previous bioassays showing the suitable virulence selected against *Ae. aegypti* adults following the procedure described for virulence determination. The primers and the cycling program used for the profiling of intron insertions verification were described above. 

The conidia strain (1 × 10^8^ conidia/mL in 0.5% INEX-A solution) was used to expose and infect *Ae. aegypti* adults in triplicate. For this, 15 mosquitoes were exposed to each strain inside a cylindrical container with 1 L capacity. The conidia suspension was dispersed on a filter paper layer that covered the container’s cylindrical part.

In addition, a cotton ball soaked in 5% sugar solution was added for feeding purposes. After two days’ exposure, mosquitoes were transferred to a new container with 250 mL capacity, which was covered with a fine mesh. Each container had enough sugar solution to ensure their feeding and survival. Survival was evaluated every 12 h for up to 15 d. According to the TL_50_, dead mosquitoes were collected from 7 d onwards for molecular strain detection (~10 mosquitoes).

Genomic DNA extraction was performed from three corpses of mosquitoes exposed to *B. bassiana* BBPTG4 strain conidia using the same conditions for intron amplification reactions as described above.

The four introns’ amplifications from DNA product bands were observed in 1% agarose gel at 119 V for 90 min, stained with ethidium bromide, and visualized in a UV trans-illuminator. BBPTG4 pure mycelia were tested as a positive control, whereas the negative control was DNA from laboratory-rearing mosquitoes, as described in Section 4.4.

## 5. Conclusions

*B. bassiana* effectively infected *Ae. aegypti* adults under laboratory conditions. Our results demonstrated that the tested strains are valuable tools for vector control management, exhibiting genetic variability based on their intron profiles. *B. bassiana* BBPTG4 strain was successfully identified in infected *Ae. aegypti* adult cadavers. To our knowledge, this is the first report of *B. bassiana* direct strain molecular identification after *Ae. aegypti* infection under laboratory conditions. Furthermore, the intron profiles indicated that insertion sites, I1P, I3P, and I4P are the targets by which to identify the BBPTG4 strain infecting *Ae. aegypti*. In addition, amplicon I1P and I3P (501 and 606 bp, respectively) served to differentiate BBPTG4 from the commercial GHA strain. These findings will allow for *B. bassiana* identification in infected mosquitoes at the strain level, its efficacy among infected mosquitoes, and its dissemination after application.

## Figures and Tables

**Table 1:** Probit regression analysis of mean time to death (LT_50_) induced by different strains of *Beauveria bassiana* against *Aedes aegypti* adults.

Strain	n ^a^	Slope ±SE	Intercept ±SE	LT_50_ Fiducial Limits ^b^ (days)	χ^2 c^
BBPTG4	10	7.30 ± 0.88	−6.39 ± 0.74	7.5 (6.96−8.04)	8.33
GHA	11	5.48 ± 0.52	−3.93 ± 0.39	5.22 (4.80−5.66)	2.53
BB42	Nd				
BB01	12	12.40 ± 1.70	−12.21 ± 1.70	9.65 (9.24–10.15)	7.60
BBPTG6	9	6.50 ± 0.84	−4.34 ± 0.58	4.65 (4.24–5.07)	5.25
BB37	8	5.27 ± 0.71	−2.45 ± 0.39	2.92 (2.55–3.29)	3.24
BB02	10	6.90 ± 0.92	−4.63 ± 0.64	4.67 (4.25–5.13)	1.97

Probit regressions were fitted using the program PoloPlus. ^a^ n = number of days analyzed. ^b^ Mean time to death. ^c^ Probit fit adequacy model. All parallelism tests were significant (*p* < 0.05). Nd = not determined.

## Data Availability

The data presented in this study are available on request from the corresponding author.

## Supplementary Materials

The supporting information can be downloaded at: https://www.mdpi.com/article/10.3390/ijms25168807/s1.

## Abbreviations

acetone = Ac; colony forming units = CFU; control = ctr; doing by yourself = DYI; ethylenediamine tetraacetic acid = EDTA; formulated microgranule = FMG; lactic acid = LA; potato dextrose agar = PDA; potato dextrose broth = PDB; ribosomal desoxyribonucleic acid = rDNA; ribosomal internal transcribed spacers = ITSs; sodium dodecyl sulfate = SDS; sucrose = Su

## References

[1] Lwande O.W., Obanda V., Lindström A., Ahlm C., Evander M., Näslund J., Bucht G. (2020). Globe-trotting *Aedes aegypti* and *Aedes albopictus*: Risk factors for arbovirus pandemics. Vector-Borne Zoonotic Dis..

[2] Secretaría de Salud Pública (SSP) Panorama Epidemiológico del Dengue. https://www.gob.mx/salud/documentos/panorama-epidemiologico-de-dengue-2024.

[3] Pan American Health Organization (PAHO). https://www3.paho.org/data/index.php/es/temas/indicadores-dengue/dengue-nacional/9-dengue-pais-ano.html.

[4] Pan American Health Organization (PAHO). https://www.gob.mx/cms/uploads/attachment/file/878914/CuadroCasosZikayEmbsem52_2023inst.pdf.

[5] Pan American Health Organization (PAHO). https://www.gob.mx/cms/uploads/attachment/file/878913/CuadroCasosyDefuncionesChiksem52INST_2023.pdf.

[6] Howard A., Guessan R., Koenraadt C., Asidi A., Farenhorst M., Akobéto M., Knols B., Takken W. (2011). First report of the infection of insecticide-resistant malaria vector mosquitoes with an entomopathogenic fungus under field conditions. Malar. J..

[7] Wang Y., Wang X., Brown D.J., An M., Xue R.D., Liu N. (2023). Insecticide resistance: Status and potential mechanisms in *Aedes aegypti*. Pestic. Biochem. Physiol..

[8] Huang Y.J.S., Higgs S., Vanlandingham D.L. (2017). Biological control strategies for mosquito vectors of arboviruses. Insects.

[9] Qin Y., Liu X., Peng G., Xia Y., Cao Y. (2023). Recent advancements in pathogenic mechanisms, applications and strategies for entomopathogenic fungi in mosquito biocontrol. J. Fungi.

[10] Cruz L.P., Gaitan A.L., Gongora C.E. (2006). Exploiting the genetic diversity of *Beauveria bassiana* for improving the biological control of the coffee berry borer through the use of strain mixtures. Appl. Microbiol. Biotechnol..

[11] Valero-Jiménez C.A., Debets A.J., van Kan J.A., Schoustra S.E., Takken W., Zwaan B.J., Koenraadt C.J. (2014). Natural variation in virulence of the entomopathogenic fungus *Beauveria bassiana* against malaria mosquitoes. Malar. J..

[12] Abboud R., Mouhanna A.M., Choueiri E., El-Rahbana B. (2012). Assessment of the effectiveness of *Beauveria bassiana* fungus in controlling insects under greenhouse, field and laboratory conditions. Pers. Gulf Crop Prot..

[13] Inglis G.D., Enkerli J.U.E.R.G., Goettel M.S. (2012). Laboratory techniques used for entomopathogenic fungi: Hypocreales. Man. Tech. Invertebr. Pathol..

[14] Gasmi L., Baek S., Kim J.C., Kim S., Lee M.R., Park S.E., Shin T.Y., Lee S.J., Parker B.L., Kim J.S. (2021). Gene diversity explains variation in biological features of insect killing fungus, *Beauveria bassiana*. Sci. Rep..

[15] Schoch C.L., Seifert K.A., Huhndorf S., Robert V., Spouge J.L., Levesque C.A., White M.M. (2012). Nuclear ribosomal internal transcribed spacer (ITS) region as a universal DNA barcode marker for Fungi. Proc. Natl. Acad. Sci. USA.

[16] Uztan A.H., Gunyar O.A., Yoltas A., Keskin N. (2016). Isolation and identification of entomopathogenic fungi *Beauveria bassiana* from Turkey. Fresenius Environ. Bull.

[17] Mitina G.V., Tokarev Y.S., Movila A.A., Yli-mattila T.T. (2011). Tick-borne diseases polymorphism of *Beauveria bassiana* (Deuteromycota: Hyphomycetes) strains isolated from *Ixodes ricinus* (Acari: Ixodidae) in Moldova. Ticks Tick-Borne Dis..

[18] Dhar S., Jindal V., Jariyal M., Gupta V.K. (2019). Molecular characterization of new isolates of the entomopathogenic fungus *Beauveria bassiana* and their efficacy against the tobacco caterpillar, *Spodoptera litura* (Fabricius) (Lepidoptera: Noctuidae). Egypt J. Biol. Pest. Cont..

[19] Kumar N.S., Gurusubramanian G. (2011). Random amplified polymorphic DNA (RAPD) markers and their applications. Sci. Vis..

[20] Castrillo L.A., Vandenberg J.D., Wraight S.P. (2003). Strain-specific detection of introduced *Beauveria bassiana* in agricultural fields by use of sequence-characterized amplified region markers. J. Invertebr. Pathol..

[21] Dutech C., Enjalbert J., Fournier E., Delmotte F., Barres B., Carlier J., Tharreau D., Giraud T. (2007). Challenges of microsatellite isolation in fungi. Fungal Genet. Biol..

[22] Dujon B. (1989). Group I introns as mobile genetic elements: Facts and mechanistic speculations—A review. Gene.

[23] Saldanha R., Mohr G., Belfort M., Lambowitz A.M. (1993). Group I and group II introns. FASEB J..

[24] Neuvéglise C., Brygoo Y., Riba G. (1997). 28S rDNA group-I introns: A powerful tool for identifying strains of *Beauveria brongniartii*. Mol. Ecol..

[25] Wang C.S., Li Z., Typas M.A., Butt T.M. (2003). Nuclear large subunit rDNA group I intron distribution in a population of *Beauveria bassiana* strains: Phylogenetic implications. Mycol. Res..

[26] Cavazos-Vallejo T., Valadez-Lira J.A., Orozco-Flores A.A., Gomez-Flores R., Ek-Ramos M.J., Quistián-Martínez D., Alcocer-González J.M., Tamez-Guerra P. (2023). In Planta detection of *Beauveria bassiana* (Ascomycota: Hypocreales) strains as endophytes in bean (*Phaseolus vulgaris* L.). Plants.

[27] Tawidian P., Kang Q., Michel K. (2023). The potential of a new *Beauveria bassiana* isolate for mosquito larval control. J. Med. Entomol..

[28] Zhang Z., Lu Y., Xu W., Sui L., Du Q., Wang Y., Li Q. (2020). Influence of genetic diversity of seventeen *Beauveria bassiana* isolates from different hosts on virulence by comparative genomics. BMC Genom..

[29] Zimmermann G. (2007). Review on safety of the entomopathogenic fungi *Beauveria bassiana* and *Beauveria brongniartii*. Biocontrol Sci. Technol..

[30] Gebremariam A., Chekol Y., Assefa F. (2021). Phenotypic, molecular, and virulence characterization of entomopathogenic fungi, *Beauveria bassiana* (Balsam) Vuillemin, and *Metarhizium anisopliae* (Metschn.) Sorokin from soil samples of Ethiopia for the development of mycoinsecticide. Heliyon.

[31] Meyling N.V., Eilenberg J. (2007). Ecology of the entomopathogenic fungi *Beauveria bassiana* and *Metarhizium anisopliae* in temperate agroecosystems: Potential for conservation biological control. Biol. Control.

[32] Landa B.B., López-Díaz C., Jiménez-Fernández D., Montes-Borrego M., Muñoz-Ledesma F.J., Ortiz-Urquiza A., Quesada-Moraga E. (2013). In-planta detection and monitorization of endophytic colonization by a *Beauveria bassiana* strain using a new-developed nested and quantitative PCR-based assay and confocal laser scanning microscopy. J. Invertebr. Pathol..

[33] de Paula A.R., Brito E.S., Pereira C.R., Carrera M.P., Samuels R.I. (2008). Susceptibility of adult *Aedes aegypti* (Diptera: Culicidae) to infection by *Metarhizium anisopliae* and *Beauveria bassiana*: Prospects for Dengue vector control. Biocontrol. Sci. Technol..

[34] de la Rosa W., Lopez F.L., Liedo P. (2002). *Beauveria bassiana* as a pathogen of the Mexican fruit fly (Diptera: Tephritidae) under laboratory conditions. J. Econ. Entomol..

[35] García-Munguía A.M., Garza-Hernández J.A., Rebollar-Tellez E.A., Rodríguez-Pérez M.A., Reyes-Villanueva F. (2011). Transmission of *Beauveria bassiana* from male to female *Aedes aegypti* mosquitoes. Parasit. Vector.

[36] Leles R.N., Sousa N.A., Rocha L.F.N., Santos A.H., Silva H.H.G., Luz C. (2010). Pathogenicity of some hypocrealean fungi to adult *Aedes aegypti* (Diptera: Culicidae). Parasitol. Res..

[37] Quesada-Moraga E., Gonzalez-Mas N., Yousef-Yousef M., Garrido-Jurado I., Fernandez-Bravo M. (2024). Key role of environmental competence in successful use of entomopathogenic fungi in microbial pest control. J. Pest. Sci..

[38] Butt T.M., Coates C.J., Dubovskiy I.M., Ratcliffe N.A. (2016). Entomopathogenic fungi: New insights into host–pathogen interactions. Adv. Genet..

[39] Jaronski S.T., Jackson M.A., Lacey L.A. (2012). Mass Production of Entomopathogenic Hypocreales. Manual of Techniques in Invertebrate Pathology.

[40] Tamayo-Mejía F., Tamez-Guerra P., Guzmán-Franco A.W., Gomez-Flores R. (2016). Developmental stage affects survival of the ectoparasitoid *Tamarixia triozae* exposed to the fungus *Beauveria bassiana*. Biol. Control..

[41] Castrejon-Antonio J.E., Nuñez-Mejia G., Iracheta M.M., Gomez-Flores R., Tamayo-Mejia F., Ocampo-Hernandez J.A., Tamez-Guerra P. (2017). *Beauveria bassiana* blastospores produced in selective medium reduce survival time of *Epilachna varivestis* Mulsant larvae. Southwest Entomol..

