# Supplementary material for: Intra-Phenotypic and -Genotypic Variations of Beauveria bassiana (Bals.) Vuill. Strains Infecting Aedes aegypti L. Adults
Source: Int J Mol Sci. 2024 Aug 13;25(16):8807. doi: 10.3390/ijms25168807 (PMC11354911; doi:10.3390/ijms25168807)
Supplement: Supplementary file 1 [file ijms-25-08807-s001.zip › ijms-3112465-supplementary.pdf]

Supplementary Table S1. Source and phenotypic characteristic of tested *Beauveria bassiana* strains[1]

Strain code / macroscopic morphology	Isolated from	Macroscopic characteristics
**BB01** 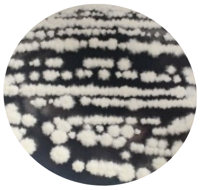	Soil	Filiform growth pattern, beige, circular shape, velvety texture, and raised elevation after 6 d culture
**BB02** 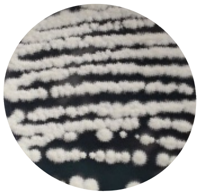	Soil	Filiform growth pattern, beige, round shape, velvety texture, and raised elevation after 6 d culture
**BB37** 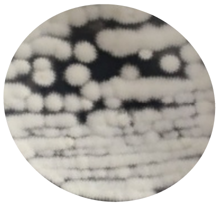	*Melanoplus differentialis* Thomas	Faster growth, filiform and dispersed growth pattern, circular shape, cottony texture, and convex elevation after 6 d culture
**BB42** 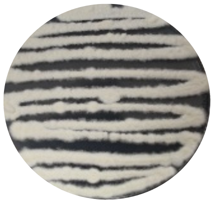	*Phyllophaga* sp.	Filiform growth pattern, beige, circular shape, velvety texture, and raised elevation after 6 d culture
**BBPTG4** 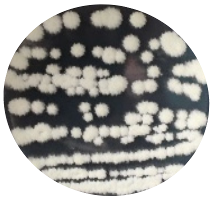	*Periplaneta americana* L.	Filiform growth pattern, circular shape, cottony texture, and convex elevation after 6 d culture
**BBPTG6** 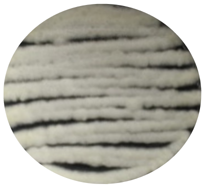	*Periplaneta americana* L.	Faster growth, filiform growth pattern, circular shape, cottony texture, and convex elevation after 6 d culture
**GHA** 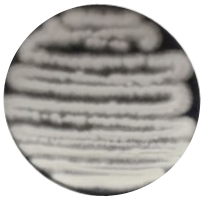	Mycotrol® commercial product	Filiform growth pattern, beige, circular shape, cottony texture, and raised elevation after 6 d culture

[1]Grown on potato dextrose agar Petri dishes, incubated at 25 °C ± 2 °C until sporulation
